# Effect of Epigallocatechin Gallate Supplementation on Inflammation, Iron Status, and Metabolomic Profiles in Women With Obesity: A Pilot Randomized Controlled Trial

**DOI:** 10.1002/mnfr.70575

**Published:** 2026-08-18

**Authors:** Sixtus Aguree, Kedir N. Turi, Alexandra R. Handt, Marian Kohut, Manju B. Reddy

**Affiliations:** ^1^ Department of Applied Health Science Indiana University School of Public Health Bloomington Indiana USA; ^2^ Department of Food Science and Human Nutrition Iowa State University Ames Iowa USA; ^3^ Department of Epidemiology and Biostatistics Indiana University School of Public Health Bloomington Indiana USA; ^4^ Department of Kinesiology Iowa State University Ames Iowa USA

**Keywords:** epigallocatechin gallate, inflammation, iron homeostasis, lipid metabolism, metabolomics, obesity

## Abstract

Obesity in women of reproductive age is often associated with low‐grade inflammation and functional iron deficiency. Epigallocatechin gallate (EGCG), a major green tea catechin, has antioxidant, anti‐inflammatory, and metabolic‐regulating properties but may also interfere with iron absorption. This randomized, double‐blind, placebo‐controlled pilot study evaluated the effects of EGCG on lipid profile, inflammation, iron status, and plasma metabolomics in women with obesity. Seventeen participants (BMI ≥30 kg/m^2^; age 20–44 years) received 400 mg/day EGCG (green tea extract: 98% polyphenols, 60% catechins, ∼50% EGCG) or placebo for eight weeks. Outcomes included serum lipids, inflammatory markers (CRP, IL‐6, TNF‐α, IL‐10), iron indices (ferritin, hepcidin, serum iron, transferrin saturation), glucose, and metabolomics. No between‐group difference remained significant after correction for multiple comparisons. Total cholesterol (β = −10.9 mg/dL; *p*  = 0.16) and LDL‐C (β = −9.7 mg/dL; *p*  = 0.16) showed decreases, and IL‐6 the largest, though nonsignificant, reduction (*p*  = 0.09); other cytokines and glucose were unchanged. Metabolomics identified 29 nominally differential metabolites enriched in lipid metabolism, plasmalogen biosynthesis, and mitochondrial β‐oxidation, none surviving FDR correction. Iron status was stable. These findings suggest metabolic effects of EGCG and support short‐term safety at 400 mg/day. Larger, longer trials with targeted metabolomics are warranted.

## Graphical abstract

In this 8‐week double‐blind pilot RCT, 400 mg/day EGCG in women with obesity produced favorable trends in cholesterol and stable iron status without significant changes in inflammation or glucose. Exploratory metabolomics suggested modulation of lipid and mitochondrial pathways, supporting safety and warranting larger trials.

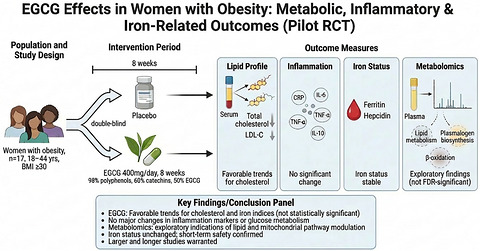

**Abbreviations**
albumin/globulin ratio: ratio of albumin to globulin
ALP: alkaline phosphatase
ALT: alanine aminotransferase
AST: aspartate aminotransferase
basophils: basophil count
bilirubin: total bilirubin concentration
BUN: blood urea nitrogen
creatinine: serum creatinine concentration
CRP: C‐reactive protein
eGFR: estimated glomerular filtration rate
eosinophils: eosinophil count
ferritin: ferritin concentration
globulin: globulin concentration in serum
glucose: blood glucose concentration
HDL: high‐density lipoprotein cholesterol
hematocrit: percentage of red blood cells in blood
hemoglobin: hemoglobin concentration
hepcidin: hepcidin concentration
IFN‐γ: interferon gamma
IL‐10: interleukin‐10
IL‐1β: interleukin‐1 beta
IL‐6: interleukin‐6
LDL: low‐density lipoprotein cholesterol
lymphocytes: lymphocyte count
MCHC: mean corpuscular hemoglobin concentration
MCV: mean corpuscular volume
monocytes: monocyte count;
non‐HDL cholesterol: total cholesterol minus HDL
RBC: red blood cell count
RDW: red blood cell distribution width
serum iron: serum iron concentration
TC: total cholesterol
TC/HDL ratio: ratio of total cholesterol to HDL
TIBC: total iron binding capacity
TNF‐α: tumor necrosis factor alpha
triglycerides: TG concentration
TSAT: transferrin saturation
WBC: white blood cell count.

## 1 Introduction

Obesity in women of reproductive age is a growing global health concern, commonly associated with low‐grade systemic inflammation, impaired iron regulation, and metabolic disturbances despite adequate nutritional intake [1]. Iron is a micronutrient of particular interest in this population. Paradoxically, despite adequate dietary intake, iron deficiency is common among obese women, a phenomenon now partly attributed to chronic, obesity‐driven inflammation. Inflammatory cytokines such as interleukin‐6 (IL‐6) and tumor necrosis factor‐alpha (TNF‐α) stimulate hepatic production of hepcidin, a regulatory peptide hormone that inhibits intestinal iron absorption and promotes macrophage iron sequestration. Elevated hepcidin levels produce functional iron deficiency even in the absence of anemia, impairing erythropoiesis, immune function, and overall well‐being [2, 3, 4, 5, 6, 7, 8].

In recent years, bioactive dietary polyphenols have received considerable attention for their potential to modulate metabolic and inflammatory processes [9, 10, 11]. Epigallocatechin gallate (EGCG), the principal catechin in green tea, is among the most extensively studied polyphenols [12, 13, 14]. Preclinical and clinical studies have demonstrated antioxidant, anti‐inflammatory, lipid‐lowering, and insulin‐sensitizing properties of EGCG [15, 16, 17, 18, 19]. At the molecular level, EGCG exerts its bioactivity through several interacting mechanisms. EGCG inhibits NF‐κB signaling and downregulates the transcription of pro‐inflammatory cytokines, thereby attenuating low‐grade inflammation. Additionally, EGCG acts as a potent reactive oxygen species (ROS) scavenger, reducing oxidative stress that is commonly elevated in obesity [20, 21]. In vitro studies have demonstrated that EGCG suppresses lipopolysaccharide‐induced secretion of TNF‐α and IL‐6 in macrophages and inhibits adipogenesis in pre‐adipocyte cell lines [15, 16]. In animal models, EGCG supplementation has been shown to reduce hepatic lipid accumulation, improve insulin sensitivity, and attenuate obesity‐associated inflammatory markers [17, 18]. At the metabolic level, EGCG activates AMP‐activated protein kinase (AMPK), a master regulator of cellular energy homeostasis that promotes fatty acid oxidation and suppresses lipogenesis [22]. EGCG also inhibits fatty acid synthase (FAS), a key enzyme in de novo lipogenesis, which may underlie its observed effects on lipid metabolism and the metabolomic shifts in β‐oxidation pathways reported here [23, 24, 25]. These mechanisms collectively position EGCG as a plausible candidate for functional food interventions targeting obesity‐related metabolic dysfunction.

An important distinction exists between purified, isolated EGCG (as used in this study) and whole green tea extracts or infusions. Whole tea preparations contain a complex matrix of polyphenols, including catechins, theaflavins, and thearubigins, as well as caffeine and other bioactive compounds, which may exert synergistic or additive effects not observed with isolated EGCG alone. Studies reporting significant anti‐inflammatory or lipid‐lowering effects have often used mixed green tea catechin extracts rather than purified EGCG, which may partly explain the more modest effects observed in trials using isolated compounds [21, 26]. In addition to its metabolic properties, EGCG possesses iron‐chelating capacity that may interfere with nonheme iron absorption [21, 26]. While such effects may be beneficial in iron‐overload conditions, they raise concerns in populations already predisposed to functional iron deficiency, including obese women with chronic, hepcidin‐mediated iron restriction. Although some studies have reported reductions in iron absorption and ferritin with green tea catechin consumption [13, 26, 27], the clinical relevance of these findings at supplemental EGCG doses and their interaction with obesity‐related inflammatory pathways remains incompletely characterized.

Previous randomized controlled trials employing EGCG doses of 400–800 mg/day for 8–12 weeks have reported modest improvements in lipid profiles and inflammatory markers, though results remain inconsistent and mechanistic evidence is limited [19, 28]. The selection of 400 mg/day for 8 weeks in the present study was informed by this literature: 400 mg/day represents the lower bound of the therapeutic dose range associated with measurable metabolic effects, and an 8‐week duration corresponds to the minimum period generally considered sufficient to detect changes in lipid or inflammatory biomarkers in clinical trials. These parameters were also chosen to maximize safety and participant compliance in this pilot context.

To date, clinical studies have rarely employed systemic metabolomic approaches to characterize the broader biochemical effects of EGCG supplementation. Untargeted metabolomics enables high‐resolution profiling of metabolic responses to dietary interventions and can reveal pathway‐level changes not captured by traditional clinical biomarkers [29, 30]. Given these gaps, the present pilot study was designed to simultaneously evaluate the effects of 8 weeks of EGCG supplementation at 400 mg/day in women with obesity on lipid metabolism, inflammatory cytokines, iron biomarkers, and untargeted plasma metabolomics.

## 2 Materials And Methods

### 2.1 Ethical Approval

The study protocol was approved by the Institutional Review Board (IRB) of Iowa State University (IRB Protocol No. 20–325). All participants provided written informed consent prior to enrollment. All procedures were conducted in accordance with the Declaration of Helsinki.

### 2.2 Study Design and Population

**FIGURE 1:**
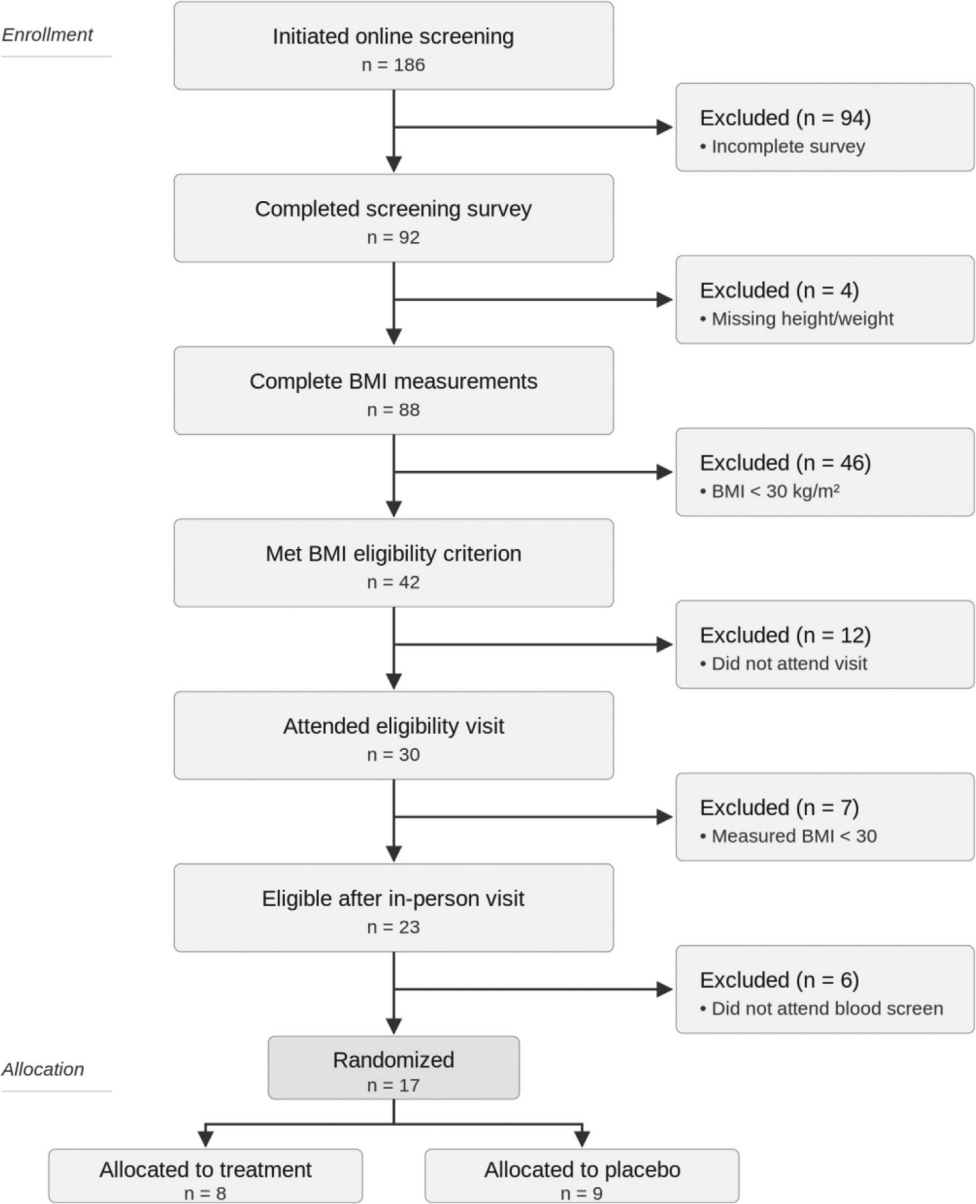
CONSORT flow diagram of participant screening, eligibility assessment, and randomization. Figure Legend: Flow diagram showing participant progression from initial screening through randomization. A total of 186 participants initiated screening, of whom 92 completed the screening survey. Participants were excluded sequentially for incomplete surveys, missing measurements, BMI <30 kg/m^2^, nonattendance at the eligibility visit, ineligibility after the visit, or failure of the blood screening criteria. Overall, 17 participants were randomized, with 8 allocated to the treatment group and 9 allocated to the placebo group. CONSORT, Consolidated Standards of Reporting Trials.

This randomized, double‐blind, placebo‐controlled, parallel‐group pilot study was conducted over an 8‐week period at the Nutrition and Wellness Research Center at Iowa State University. The study was conducted between October 2021 and April 2023. Recruitment was conducted through flyers, online posts, and University listings. Eligible participants were women aged 20–44 years with a body mass index (BMI) between 30.0 and 39.9 kg/m^2^ [31]. Of 186 individuals who initiated the online screening form, 92 completed it in full. Of these, 88 self‐reported height and weight; 42 had a self‐reported BMI between 30 and 40 kg/m^2^ and were invited to an in‐person screening visit to verify BMI and eligibility. Thirty attended the in‐person screening, 23 met all eligibility criteria, and 17 completed blood screening and were randomized to the treatment (n = 8) or placebo (n = 9) arm (Figure 1—CONSORT Flow Diagram).

Exclusion criteria included pregnancy or breastfeeding; hemoglobin < 7.0 g/dL; current smoking; recent antibiotic use; alcohol consumption > 14 drinks/week; or use of iron supplements, antioxidants, lipid‐lowering drugs, or anti‐inflammatory supplements within the past month. Additional exclusion criteria included a diagnosis of diabetes, cardiovascular, gastrointestinal, or hematologic disease, or engagement in high‐intensity physical activity ≥4 times per week. Eligibility was confirmed through online screening followed by an in‐person review including anthropometric measurements and blood chemistry analysis.

### 2.3 Randomization, Blinding, and Intervention

Participants were randomized 1:1 to receive EGCG or placebo using a computer‐generated block randomization schedule stratified by age and BMI. Allocation was concealed in sequentially numbered opaque envelopes prepared by a researcher not involved in data collection or outcome assessment.

Participants in the intervention group received 400 mg/day of a highly purified green tea leaf extract (*Camellia sinensis* [L.] Kuntze) containing 98% polyphenols (60% catechins), of which approximately 50% was EGCG, generously supplied by Givaudan (South Hackensack, NJ, USA). The extract was tested by the manufacturer for heavy metals and microbial contaminants prior to use, and its polyphenol composition was verified by high‐performance liquid chromatography (HPLC). The supplement was delivered in the form of two 200 mg capsules taken together once daily in the morning with water, providing a total polyphenol dose of approximately 784 mg/day, of which ∼400 mg was EGCG. The placebo group received identical‐appearing capsules containing microcrystalline cellulose, purchased from the Allergy Research Group (distributed by PureFormulas, Miami Lakes, FL, USA). Capsules were prepared under standardized conditions at the metabolic kitchen unit.

Participants were instructed to maintain their habitual diet and physical activity throughout the study and to abstain from caffeine‐containing products, green tea, and dietary supplements. To minimize potential confounding from polyphenol‐rich foods on the metabolomic data, participants were also instructed to limit intake of foods high in polyphenols including berries, dark chocolate, and high‐polyphenol beverages (e.g., coffee, red wine) during the study period. Participants recorded their dietary intake using standardized 3‐day food records (two weekdays and one weekend day) at both baseline and week 8. These records were analyzed using validated dietary analysis software to assess macronutrient composition, energy intake, and compliance with polyphenol and caffeine restrictions (Table S1). Body weight was measured at baseline and at week 8 to monitor for unintentional weight change that could independently alter metabolic or inflammatory outcomes.

Compliance with capsule intake was monitored via capsule counts returned at each study visit. Adherence to caffeine and green tea restrictions was primarily self‐reported. Participants who were < 80% compliant by capsule count were to be excluded from the analysis; all 17 participants exceeded this threshold.

### 2.4 Outcome Measures

Primary outcomes were changes in traditional lipid biomarkers (total cholesterol, LDL‐C, HDL‐C, and triglycerides), inflammatory cytokines (IL‐6, IL‐1β, IL‐10, TNF‐α, IFN‐γ, and hs‐CRP), and iron‐related biomarkers (hemoglobin, MCV, serum iron, ferritin, hepcidin, transferrin saturation, and total iron binding capacity). Blood samples were collected at baseline and at week 8. Secondary outcomes included untargeted metabolomic analysis of plasma to identify changes in biochemical pathways. All blood samples were collected between 08:00 and 10:00 h following an overnight fast.

### 2.5 Blood Collection and Laboratory Analysis

Venous blood was collected and processed within 2 h of collection; plasma and serum were aliquoted and stored at −80°C until analysis. Samples were analyzed by a certified clinical laboratory (Quest Diagnostics, Chicago, IL, USA) for comprehensive biochemical and hematological panels, including lipid profiles, blood chemistry, liver function markers, hematologic indices, and the iron status panel (serum iron, total iron binding capacity [TIBC], and transferrin saturation [TSAT]).

Hepcidin‐25 (bioactive) was quantified by ELISA (DRG International, Inc., Springfield, NJ, USA; Cat# EIA5782R). Serum ferritin was measured using an ELISA kit from ALPCO Diagnostics (Salem, NH, USA; Cat# 11‐FERHU‐E01). CRP was measured with a kit from ALPCO Diagnostics (Cat# 30–9710S). All ELISA assays were run in duplicate per manufacturer instructions using a microplate reader (Bio‐Tek Instruments Inc., Winooski, VT, USA). Inflammatory cytokines (IL‐1β, IL‐6, IL‐10, TNF‐α, IFN‐γ) were quantified by high‐sensitivity multiplex immunoassay (MILLIPLEX Human High Sensitivity T Cell Panel, MilliporeSigma, Burlington, MA, USA) using Luminex xMAP technology. Assay sensitivity ranged from 0.11 to 8.17 pg/mL, with intra‐ and inter‐assay coefficients of variation below 10% and 15%, respectively.

Untargeted metabolomic profiling of fasting plasma samples (n = 28) was performed using high‐resolution liquid chromatography–mass spectrometry (LC‐MS). Samples underwent methanol‐based protein precipitation, sonication at 4°C, incubation, and centrifugation. The supernatant was transferred to LC‐MS vials and spiked with DL‐o‐chlorophenylalanine as an internal standard. Quality control (QC) samples were prepared from pooled extracts and processed in parallel. Analyses used a Vanquish Flex UPLC coupled with a Q Exactive Plus Orbitrap MS (Thermo Fisher Scientific) with chromatographic separation on an ACQUITY UPLC HSS T3 column (100 × 2.1 mm, 1.8 µm). Data were processed using Compound Discoverer v3.0 for feature extraction and normalization, and multivariate statistical modeling was performed using SIMCA‐P v14.1 (Umetrics).

### 2.6 Statistical Analysis

Descriptive statistics are expressed as mean ± SD, geometric mean (95% CI) for right‐skewed variables, or frequency (%). Linear mixed‐effects models with fixed effects for treatment, time, and their interaction and a random intercept for participant were used for biomarker analyses. In these models the treatment × time interaction is the coefficient of interest: it estimates the difference between groups in the average change from baseline to week 8 (the mean change under EGCG minus the mean change under placebo), so that a value different from zero indicates the outcome changed differently in the two arms. With two timepoints this interaction is equivalent to a difference‐in‐differences—each participant's week‐8‐minus‐baseline change, compared between arms. For each model, the treatment × time interaction was also expressed as a standardized coefficient (the interaction estimated on the z‐scored outcome) to provide a Cohen's d–type effect size. Right‐skewed variables (triglycerides, AST, ALT, the inflammatory cytokines, ferritin, hepcidin, and CRP) were summarized as geometric means (95% CI) and modeled on the natural‐log scale. Within‐group changes were assessed by paired t‐tests. Given the pilot and exploratory nature of this study, no adjustments for multiple comparisons were applied to the clinical biomarker analyses. As sensitivity analyses, the between‐group treatment effect was re‐estimated for every outcome using baseline‐adjusted models: analysis of covariance (the week‐8 value regressed on treatment and the baseline value) and a constrained longitudinal data analysis that retains participants with a missing baseline value.

Metabolomic features were normalized and log‐transformed, then visualized using principal component analysis (PCA) for quality assessment and outlier detection. Supervised models, including Partial Least Squares Discriminant Analysis (PLS‐DA) and Orthogonal PLS‐DA (OPLS‐DA), were used to identify treatment‐related features [32]. Features with a Variable Importance in Projection (VIP) score > 1.5 and unadjusted p < 0.05 were considered candidate biomarkers. Pathway enrichment analysis was performed using MetaboAnalyst 5.0 with reference to the KEGG and HMDB databases [33, 34]. Differential expression analysis applied thresholds of fold change > 2 and unadjusted p < 0.05. Benjamini–Hochberg FDR correction was subsequently applied to all metabolomic comparisons; no metabolite features remained significant after correction, confirming that all metabolomic findings are exploratory. All analyses were performed using Stata 19.5 (StataCorp, College Station, TX) and R v4.5.1 for the metabolomics [35].

## 3 RESULTS

### 3.1 Participant Characteristics and Compliance

Seventeen women with obesity (BMI 30–40 kg/m^2^), all self‐identified as White and non‐Hispanic, completed the 8‐week RCT and were included in the final analysis. Participants were assigned to the EGCG group (n = 8) or the placebo group (n = 9), with no dropouts or protocol violations reported (Figure 1). The majority were university students (n = 10). Baseline characteristics including age (mean = 26.5 ± 9.1 years) and BMI (34.0 ± 3.3 kg/m^2^) were similar between groups, although age was marginally higher in the placebo group (*p* = 0.050; Table 1). No significant change in body weight during the study (*p*>0.90). Dietary intake estimated from 3‐day food records did not differ significantly between groups in energy or any macronutrient at either baseline or week 8, and micronutrient intake was largely comparable, with the exception of a nominally lower vitamin A intake in the EGCG group at week 8 (Table S1). Intervention adherence exceeded 90% in both groups as confirmed by capsule counts. No adverse events were reported. These results confirm the short‐term safety and feasibility of 400 mg/day EGCG supplementation in young women with obesity, as evidenced by the absence of adverse events, high adherence, and stable safety laboratory parameters throughout the trial.

**TABLE 1:** Baseline biochemical, hematologic, and inflammatory variables by treatment group^1^.

Variable	Reference range or cut‐off	Total (n = 17)	Placebo (n = 9)	EGCG (n = 8)	*p*
***Demographics***					
Age (years)	—	26.5 (9.1)	30.6 (10.9)	22.0 (3.3)	0.050
BMI (kg/m^2^)	18.5–24.9	34.0 (3.3)	34.0 (3.5)	33.9 (3.2)	0.96
***Lipid Panel***					
Total cholesterol (mg/dL)	<200	184.2 (25.9)	190.9 (31.1)	176.8 (17.5)	0.27
HDL cholesterol (mg/dL)	>50	53.7 (12.5)	58.0 (15.4)	48.9 (5.9)	0.14
Triglycerides (mg/dL)	<150	100.1 (81.8–122.5)	96.6 (68.6–136.1)	104.2 (77.7–139.5)	0.71
*ln(Triglycerides)*		*4.61 (0.10)*	*4.57 (0.15)*	*4.65 (0.12)*	
LDL cholesterol (mg/dL)	<100	109.9 (23.9)	112.3 (31.8)	107.1 (11.6)	0.67
TC/HDL ratio	<5.0	3.5 (0.7)	3.5 (0.9)	3.6 (0.3)	0.68
Non‐HDL cholesterol (mg/dL)	<130	130.5 (25.4)	132.9 (33.2)	127.9 (13.9)	0.70
***Metabolic Panel***					
Glucose (mg/dL)	65–99	91.3 (6.5)	90.0 (8.3)	92.8 (3.6)	0.40
BUN (mg/dL)	7–25	12.1 (3.2)	13.2 (3.6)	10.9 (2.2)	0.13
Creatinine (mg/dL)	0.50–1.05	0.75 (0.10)	0.75 (0.12)	0.74 (0.08)	0.87
eGFR (mL/min/1.73 m^2^)	≥60	110.4 (15.2)	107.3 (18.5)	113.8 (10.6)	0.40
Albumin (g/dL)	3.6–5.1	4.5 (0.3)	4.5 (0.3)	4.5 (0.3)	0.48
Globulin (g/dL)	1.9–3.7	2.7 (0.3)	2.7 (0.2)	2.7 (0.3)	0.86
Albumin/Globulin ratio	1.0–2.5	1.7 (0.2)	1.6 (0.1)	1.7 (0.3)	0.38
Total bilirubin (mg/dL)	0.2–1.2	0.6 (0.2)	0.5 (0.2)	0.6 (0.3)	0.74
ALP (IU/L)	37–153	69.6 (12.4)	65.9 (12.9)	73.9 (11.1)	0.19
AST (IU/L)	10–35	15.9 (10.8–23.6)	13.4 (10.6–16.9)	19.4 (7.9–47.3)	0.33
*ln(AST)*		*2.77 (0.19)*	*2.59 (0.10)*	*2.96 (0.38)*	
ALT (IU/L)	6–29	15.7 (11.6–21.1)	13.4 (11.0–16.3)	18.7 (9.7–36.2)	0.25
*ln(ALT)*		*2.75 (0.14)*	*2.59 (0.08)*	*2.93 (0.28)*	
***Hematology (n = 16; Placebo n = 8)***					
WBC (×10^3^/µL)	3.8–10.8	7.0 (1.9)	7.2 (1.9)	6.8 (2.0)	0.69
RBC (×10^6^/µL)	3.80–5.10	4.7 (0.3)	4.6 (0.2)	4.7 (0.3)	0.30
Hemoglobin (g/dL)	11.7–15.5	13.6 (0.8)	13.4 (0.8)	13.8 (0.8)	0.40
Hematocrit (%)	35–45	40.0 (2.0)	39.5 (2.2)	40.4 (1.9)	0.42
MCV (fL)	80–100	85.9 (3.2)	86.3 (3.7)	85.6 (2.9)	0.68
MCHC (g/dL)	32–36	34.0 (0.8)	33.9 (0.7)	34.0 (0.9)	0.67
RDW (%)	11.0–15.0	12.5 (0.5)	12.4 (0.4)	12.6 (0.6)	0.51
Neutrophils (×10^3^/µL)	1.5–7.8	3.90 (1.39)	3.83 (1.49)	3.96 (1.37)	0.86
Lymphocytes (×10^3^/µL)	0.85–3.9	2.44 (0.79)	2.66 (0.89)	2.22 (0.65)	0.28
Monocytes (×10^3^/µL)	0.2–0.95	0.52 (0.18)	0.54 (0.15)	0.51 (0.21)	0.81
Eosinophils (×10^3^/µL)	0.015–0.50	0.106 (0.089)	0.139 (0.111)	0.072 (0.045)	0.14
Basophils (×10^3^/µL)	0.0–0.2	0.041 (0.015)	0.043 (0.014)	0.039 (0.017)	0.65
***Iron Status and Inflammatory Markers***					
Serum iron (µg/dL)	40–190	99.2 (42.0)	104.9 (45.3)	92.8 (40.0)	0.57
TIBC (µg/dL)	250–450	368.4 (54.0)	382.6 (48.0)	352.5 (59.1)	0.27
TSAT (%)	16–45	25.8 (13.8)	28.1 (13.0)	23.2 (15.1)	0.49
IFN‐γ (pg/mL)	—	25.5 (17.1–38.1)	20.8 (9.8–44.4)	31.1 (19.6–49.4)	0.30
*ln(IFN‐γ)*		*3.24 (0.19)*	*3.04 (0.32)*	*3.44 (0.20)*	
IL‐10 (pg/mL)	—	22.0 (12.5–38.6)	25.1 (10.3–61.4)	19.6 (7.8–49.4)	0.65
*ln(IL‐10)*		*3.09 (0.26)*	*3.22 (0.37)*	*2.97 (0.39)*	
IL‐1β (pg/mL)	—	2.5 (1.8–3.5)	2.4 (1.3–4.5)	2.6 (1.7–4.2)	0.78
*ln(IL‐1β)*		*0.92 (0.16)*	*0.87 (0.27)*	*0.96 (0.19)*	
IL‐6 (pg/mL)	—	2.4 (0.9–6.2)	2.2 (0.4–12.3)	2.7 (0.7–9.8)	0.82
*ln(IL‐6)*		*0.88 (0.44)*	*0.78 (0.73)*	*0.99 (0.55)*	
TNF‐α (pg/mL)	—	4.4 (3.6–5.6)	4.2 (2.8–6.2)	4.7 (3.5–6.5)	0.58
*ln(TNF‐α)*		*1.49 (0.10)*	*1.43 (0.17)*	*1.55 (0.13)*	
Ferritin (ng/mL)	10–800	20.7 (11.1–38.4)	21.1 (9.3–47.8)	20.1 (6.1–66.2)	0.94
*ln(Ferritin)*		*3.03 (0.29)*	*3.05 (0.35)*	*3.00 (0.50)*	
Hepcidin (ng/mL)	0.35–80	4.0 (2.1–7.6)	3.9 (1.7–9.3)	4.1 (1.2–13.9)	0.96
*ln(Hepcidin)*		*1.38 (0.30)*	*1.37 (0.38)*	*1.40 (0.52)*	
CRP (mg/L)	<3.0	4.8 (3.4–6.8)	4.9 (2.9–8.4)	4.7 (2.6–8.4)	0.86
*ln(CRP)*		*1.57 (0.16)*	*1.60 (0.23)*	*1.54 (0.25)*	

^1^ Data are mean (SD) for normally distributed variables and geometric mean (95% CI) for right‐skewed variables, with the natural‐log mean (SE) shown in italics below each. Between‐group comparisons used independent t‐tests (on the log scale for skewed variables). For skewed variables the p‐value is shown once, on the geometric‐mean row, and applies equally to the natural‐log (mean, SE) row beneath it, which presents the same comparison. Hematology n = 16 (Placebo n = 8); baseline cytokines n = 16 (IL‐10 n = 15) owing to missing samples. One participant had markedly elevated baseline transaminases (AST 267, ALT 252 IU/L), retained here; excluding this participant, baseline AST and ALT geometric means were 13.3 and 13.8 IU/L. Reference ranges for the lipid, metabolic, hematology, and iron panels are the performing laboratory's (Quest Diagnostics) adult‐female intervals; the ferritin range (10–800 ng/mL) is the ALPCO ELISA (11‐FERHU‐E01) measuring range; the CRP cut‐off is the AHA/CDC cardiovascular‐risk threshold (<3.0 mg/L); the hepcidin range is the DRG Hepcidin 25 ELISA measuring range; the multiplex (MILLIPLEX) cytokines have no harmonized clinical reference interval. Entries shown with ‘<’, ‘>’, or ‘≥’ are clinical decision cut‐offs or desirable targets (lipids, eGFR, CRP) rather than population reference intervals. Age was marginally higher in the placebo group (p = 0.050); all other baseline comparisons were nonsignificant.

### 3.2 Lipid Profile

After eight weeks of supplementation, modest nonsignificant reductions in total and LDL cholesterol were observed in the EGCG group. Total cholesterol: *β* = −10.92 mg/dL (95% CI −26.30 to 4.47; *p* = 0.16); LDL cholesterol: *β* = −9.68 mg/dL (95% CI −23.33 to 3.97; *p* = 0.17). No significant differences were observed for HDL cholesterol (*β* = 0.04 mg/dL; *p* = 0.99) or triglycerides (modeled on the natural‐log scale; *β* = −0.10 log‐units; 95% CI −0.26 to 0.07; *p* = 0.24) (Table 2). These directional trends are consistent with the lipid‐lowering potential of EGCG but require confirmation in larger, adequately powered trials.

**TABLE 2:** Treatment × time interaction effects on clinical and laboratory parameters^1^.

Outcome	B (SE)	95% CI	*Std. β*	*p*
***Lipids and Glucose***				
Total cholesterol	−10.92 (7.85)	−26.30, 4.47	−0.38	0.16
HDL cholesterol	0.04 (2.50)	−4.85, 4.93	0.00	0.99
ln(Triglycerides)	−0.10 (0.08)	−0.26, 0.07	−0.26	0.24
LDL cholesterol	−9.68 (6.97)	−23.33, 3.97	−0.40	0.17
TC/HDL ratio	−0.09 (0.16)	−0.41, 0.23	−0.13	0.58
Non‐HDL cholesterol	−10.96 (6.85)	−24.38, 2.46	−0.44	0.11
Glucose	−1.96 (2.98)	−7.81, 3.89	−0.33	0.51
***Metabolic Panel***				
BUN	2.24 (1.02)	0.24, 4.23	0.81	0.028
Creatinine	−0.01 (0.03)	−0.06, 0.04	−0.09	0.75
eGFR	0.69 (3.93)	−7.01, 8.40	0.05	0.86
Albumin	−0.19 (0.10)	−0.39, −0.00	−0.73	0.049
Globulin	−0.21 (0.11)	−0.42, 0.01	−0.63	0.059
Albumin/Globulin ratio	0.09 (0.08)	−0.06, 0.23	0.35	0.26
Total bilirubin	−0.10 (0.08)	−0.24, 0.05	−0.43	0.20
ALP	2.61 (6.89)	−10.90, 16.12	0.15	0.71
ln(AST)	−0.17 (0.20)	−0.56, 0.21	−0.28	0.38
ln(ALT)	−0.03 (0.17)	−0.36, 0.29	−0.05	0.84
***Hematology***				
WBC	0.42 (0.83)	−1.21, 2.04	0.24	0.62
RBC	−0.11 (0.10)	−0.31, 0.09	−0.40	0.30
Hemoglobin	−0.24 (0.27)	−0.77, 0.30	−0.33	0.39
Hematocrit	−0.38 (0.83)	−2.01, 1.24	−0.20	0.64
MCV	0.52 (0.83)	−1.10, 2.14	0.17	0.53
MCHC	−0.27 (0.40)	−1.05, 0.52	−0.34	0.51
RDW	0.23 (0.43)	−0.61, 1.07	0.32	0.60
Neutrophils	−0.12 (0.56)	−1.22, 0.97	−0.10	0.82
Lymphocytes	0.41 (0.31)	−0.19, 1.01	0.56	0.18
Monocytes	0.08 (0.08)	−0.07, 0.23	0.54	0.28
Eosinophils	0.01 (0.01)	−0.02, 0.04	0.14	0.41
Basophils	0.01 (0.01)	−0.00, 0.03	0.75	0.15
***Iron Metabolism and Inflammation***				
Serum iron	1.11 (20.10)	−38.29, 40.51	0.03	0.96
TIBC	−18.18 (11.59)	−40.89, 4.53	−0.33	0.12
TSAT	5.40 (6.49)	−7.32, 18.13	0.43	0.41
ln(IFN‐γ)	−0.06 (0.12)	−0.29, 0.18	−0.08	0.62
ln(IL‐10)	−0.06 (0.11)	−0.28, 0.15	−0.06	0.57
ln(IL‐1β)	0.01 (0.22)	−0.42, 0.44	0.02	0.96
ln(IL‐6)	−0.62 (0.37)	−1.34, 0.10	−0.40	0.09
ln(TNF‐α)	−0.07 (0.11)	−0.29, 0.15	−0.17	0.55
ln(Ferritin)	−0.24 (0.34)	−0.90, 0.41	−0.24	0.47
ln(Hepcidin)	−0.38 (0.35)	−1.05, 0.30	−0.34	0.28
ln(CRP)	−0.17 (0.20)	−0.57, 0.22	−0.25	0.39

^1^ Values are the treatment × time interaction coefficient B (SE) from linear mixed models with a random intercept for participant; Std. β is the standardized coefficient (Cohen's d metric). Skewed outcomes were modeled on the natural‐log scale (denoted ln). Two interaction terms (BUN and albumin) reached nominal significance (p < 0.05); given 41 unadjusted comparisons, this is consistent with chance, and none survived correction for multiple comparisons. In baseline‐adjusted sensitivity analyses (ANCOVA and a constrained longitudinal model), neither BUN nor albumin remained significant (all p ≥ 0.067), indicating that these nominal signals were sensitive to baseline imbalance.

### 3.3 Iron Status and Hematologic Indices

Iron status markers remained largely stable during the intervention. Ferritin and hepcidin (both modeled on the natural‐log scale) showed nonsignificant downward trends in the EGCG group compared to placebo (ferritin: *β* = −0.24 log‐units; p = 0.47; hepcidin: *β* = −0.38 log‐units; *p* = 0.28). Other iron indices—serum iron (*β* = 1.11 µg/dL; p = 0.96), transferrin saturation (*β* = 5.40%; p = 0.41), and TIBC (*β* = −18.18 µg/dL; *p* = 0.12)—showed no statistically significant differences between groups (Table 2). Hematologic parameters including hemoglobin, hematocrit, MCV, MCH, and MCHC were stable across groups (e.g., hemoglobin: *β* = −0.24 g/dL; *p* = 0.39).

### 3.4 Inflammatory Markers and Glucose Metabolism

Inflammatory cytokines showed nonsignificant treatment × time interactions, all modeled on the natural‐log scale. TNF‐α: *β* = −0.07 log‐units (*p* = 0.55); hs‐CRP: *β* = −0.17 log‐units (95% CI −0.57 to 0.22; *p* = 0.39); IFN‐γ: *β* = −0.06 log‐units (p = 0.62); IL‐10: *β* = −0.06 log‐units (*p* = 0.57). The largest change was a nonsignificant decrease in IL‐6 (*β* = −0.62 log‐units; 95% CI −1.34 to 0.10; *p* = 0.09) (Table 2). Fasting blood glucose was not significantly altered (*β* = −1.96 mg/dL; *p* = 0.51).

Within‐group analyses (paired baseline‐to‐week‐8 comparisons within each arm) reached significance only for serum creatinine, which rose modestly in both the placebo (+0.06 mg/dL; *p* = 0.018) and EGCG (+0.06 mg/dL; *p* = 0.003) groups, with a corresponding decline in estimated GFR (placebo −8.4 mL/min/1.73 m^2^, *p* = 0.032; EGCG −7.8 mL/min/1.73 m^2^, p = 0.006). Because these changes were of similar magnitude in both arms, produced no between‐group difference (Table 2), and left all values within the normal range, they reflect a nontreatment‐related time effect rather than an action of EGCG. Alkaline phosphatase and MCHC increased significantly in the placebo arm only, and no inflammatory cytokine or iron marker changed significantly within either group; in particular, IL‐6 rose in the placebo arm (+74%) but not in the EGCG arm (−7%), with neither change reaching significance.

### 3.5 Untargeted Metabolomics

**FIGURE 2:**
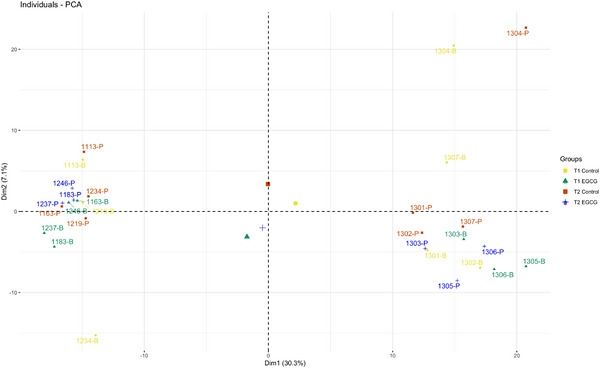
Principal component analysis (PCA) of metabolomic profiles by treatment and timepoint. PCA score plots show the distribution of individual metabolomic profiles. Samples are colored by treatment assignment and timepoint. No distinct clustering by treatment or time was observed, suggesting the absence of a strong intervention or time‐based effect and potential technical or batch‐related variation.

**FIGURE 4:**
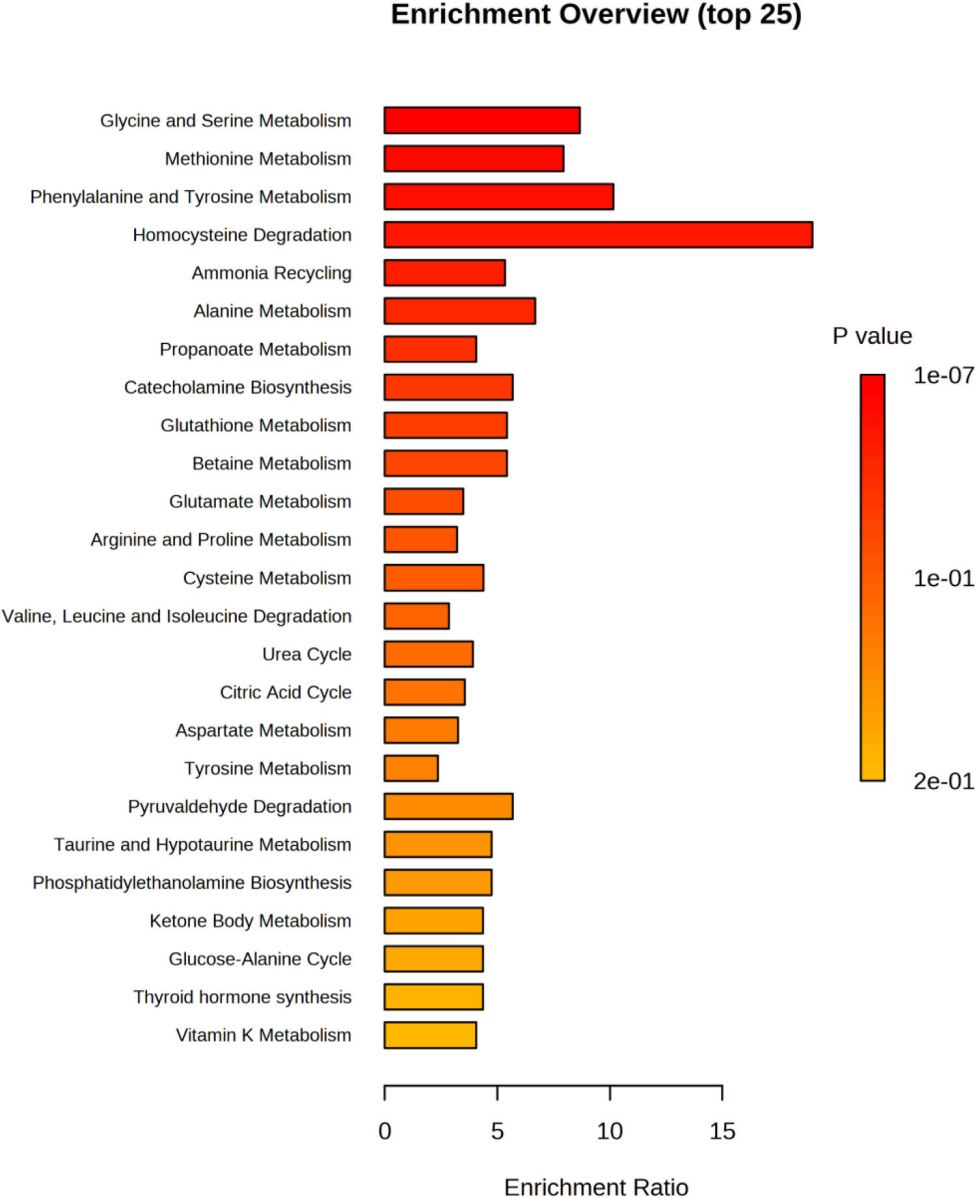
Pathway overrepresentation analysis based on unadjusted mixed model results. Pathways enriched using metabolites identified as significant (unadjusted p < 0.05) in the linear mixed model. Bars represent –log10 transformed p‐values for enrichment. These results are exploratory, as they rely on uncorrected metabolite inputs.

PCA revealed no distinct clustering by treatment group or time point, suggesting subtle rather than large‐scale metabolomic shifts (Figure 2). Supervised models (PLS‐DA, OPLS‐DA) identified potential treatment‐ or time‐dependent features. Unadjusted univariate analyses identified 29 metabolite features with nominal p < 0.05 and fold change > 2 between baseline and week 8, associated with plasmalogen biosynthesis, mitochondrial β‐oxidation, phospholipid metabolism, and arachidonic acid turnover (Table 3; Figures 3 and 4). These results are exploratory only. None of the 29 metabolite features remained statistically significant after FDR correction (Benjamini–Hochberg), and all p‐values reported for metabolomic analyses are unadjusted. The metabolomic findings should therefore be regarded as hypothesis‐generating and require confirmation in future targeted studies.

**TABLE 3:** Metabolites identified as nominally significant in linear mixed models (p < 0.05).

Metabolite ID	Putative Metabolite Name	Time Effect (p‐value)	Treatment Effect (p‐value)	Interaction (Time × Treatment) (p‐value)	Notes
M154_T10	Unknown	0.037	0.041	0.022	Detected via LC‐MS; unannotated
M207_T15	Carnitine derivative	0.044	0.031	0.028	Possible lipid metabolism link
M132_T05	Phenylalanine‐like	0.049	0.046	0.035	Aromatic amino acid pathway
M189_T09	Acylcarnitine	0.039	0.043	0.029	Medium‐chain fatty acid oxidation
M120_T03	Unknown	0.041	0.050	0.040	Not mapped to KEGG
M142_T07	Glycerophosphocholine	0.028	0.037	0.033	Membrane lipid component

FIGURE 3(A) Volcano plot of within‐person timepoint comparison (paired t‐test). Each point represents a metabolite. The x‐axis shows log2 fold‐change (Time 2 vs. Time 1), and the y‐axis displays the –log10 p‐value. Red points indicate metabolites with nominal p < 0.05 and absolute fold‐change ≥ 2. No metabolites remained significant after FDR adjustment. (B) Volcano Plot of Between‐Person Treatment Comparison (Unpaired t‐test). This volcano plot shows differential metabolite expression between treatment and control groups. Points with nominal p < 0.05 and absolute fold‐change ≥ 2 are highlighted in red. No metabolites met the FDR threshold for statistical significance.

**Figure:**
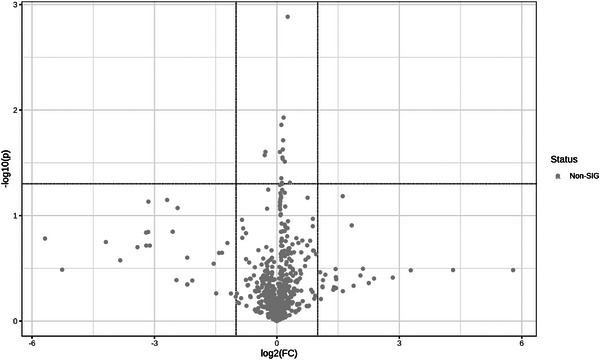

**Figure:**
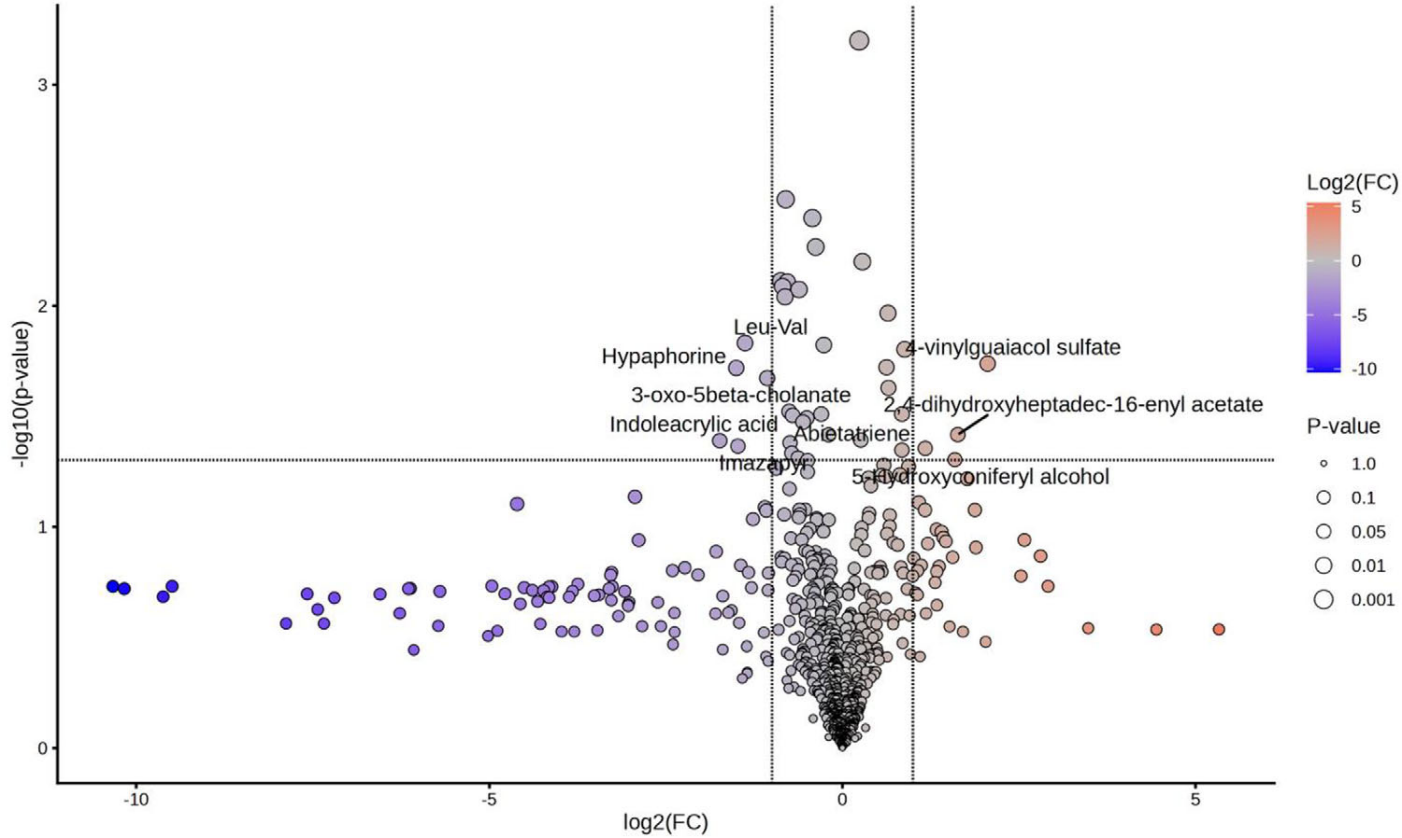

## 4 Discussion

In this randomized, double‐blind, placebo‐controlled pilot study, we investigated the effects of 8 weeks of EGCG supplementation at 400 mg/day on lipid metabolism, inflammation, iron status, and metabolomic profiles in women with obesity. While no clinical biomarker remained significant after accounting for the number of comparisons (two interaction terms, BUN and albumin, reached nominal significance at *p* < 0.05, consistent with chance among the 41 unadjusted comparisons, and neither remained significant after baseline adjustment), exploratory trends suggested biologically plausible metabolic effects of EGCG, and untargeted metabolomics revealed nominally significant pathway‐level shifts. These findings are interpreted against a growing body of evidence examining the metabolic effects of purified green tea catechins in human populations.

Consistent with prior studies in individuals with metabolic syndrome and type 2 diabetes, we observed nonsignificant reductions in total and LDL cholesterol with EGCG, while HDL and triglycerides remained unchanged. These directional lipid trends are consistent with previous trials using comparable EGCG doses (225–300 mg/day over 8–12 weeks). The magnitude of the metabolic response to EGCG depends on both dose and duration. Previous RCTs have demonstrated that supplementation at doses ≥ 400–800 mg/day for ≥ 8–12 weeks is generally required to achieve statistically significant lipid or inflammatory biomarker changes [18, 28, 36, 37, 38, 39].

Although our 8‐week protocol falls at the lower boundary of this effective range, our sample size (n = 17) provided insufficient statistical power to detect the modest effects expected at this dose; the observed effect sizes and confidence intervals are consistent with a true biological effect that larger trials could confirm. Earlier work from our groups and others reported selective reductions in serum amyloid A without changes in CRP or IL‐6 [28, 40], suggesting biomarker‐specific rather than broad anti‐inflammatory effects, a pattern consistent with our findings.

Inflammatory cytokines (TNF‐α, IL‐6, IL‐10, hs‐CRP) showed modest nonsignificant decreases in the EGCG group. This is consistent with the emerging understanding that purified, isolated EGCG, as distinct from whole green tea extracts, exerts limited systemic anti‐inflammatory effects at clinically relevant doses [41, 42]. Purified EGCG lacks the synergistic polyphenolic matrix present in whole tea preparations. Studies reporting significant reductions in CRP and IL‐6 have typically used mixed catechin extracts or EGCG combined with other bioactive compounds such as coconut oil, [41, 42] suggesting that cofactors or polyphenol synergism may be required for robust anti‐inflammatory effects. It is also plausible that the mild, stable baseline inflammatory status of our participants, relatively healthy, community‐dwelling women, limited the capacity to detect meaningful cytokine shifts, as anti‐inflammatory effects are often more pronounced in populations with higher baseline inflammation [43, 44].

The exploratory metabolomic findings represent the most novel contribution of this study. We identified 29 nominally differentially regulated metabolites (unadjusted *p* < 0.05, fold change > 2) associated with pathways central to lipid remodeling, mitochondrial β‐oxidation, plasmalogen biosynthesis, and arachidonic acid turnover (Table 3 and Figure 4). These pathway‐level shifts are consistent with two principal molecular mechanisms of EGCG action: (1) AMPK activation, which promotes fatty acid β‐oxidation and suppresses de novo lipogenesis [43, 44, 45]; and (2) inhibition of fatty acid synthase (FAS), which reduces the synthesis of fatty acids and may redirect substrate flow toward lipid remodeling pathways, including plasmalogen synthesis [46, 47]. These mechanistic interpretations are consistent with emerging omics‐based evidence showing that EGCG induces significant metabolomic shifts, including changes in lipid and phospholipid metabolism, even in the absence of detectable changes in conventional clinical markers [48, 49]. It is important to emphasize, however, that none of these metabolite features survived FDR correction, and all metabolomic results must be considered exploratory and hypothesis‐generating. These findings identify candidate pathways for future targeted metabolomic and mechanistic studies but cannot be interpreted as definitive evidence of EGCG‐induced metabolic reprogramming.

Iron status remained stable throughout the 8‐week intervention, with nonsignificant downward trends in ferritin and hepcidin but no changes in serum iron, TIBC, or transferrin saturation. These results suggest that EGCG at 400 mg/day does not materially impair systemic iron availability in women with obesity over 8 weeks—a clinically important safety finding given EGCG's known iron‐chelating capacity. This finding should be contextualized within the broader literature. Studies examining high‐dose green tea extracts or habitual tea consumption have reported reductions in serum ferritin in premenopausal and postmenopausal women and in patients with thalassemia [50, 51]. These effects have typically been attributed to the combined action of multiple catechins and other polyphenols in whole tea matrices, which collectively have greater iron‐chelating activity than isolated EGCG at equivalent doses. The supplement used in this study provided a highly purified EGCG‐enriched extract rather than a whole tea preparation, and supplementation was limited to 400 mg/day (EGCG) taken in capsule form, not as a tea infusion consumed with meals, which would maximize coingestion with dietary iron. Under these controlled conditions, the absence of iron status impairment is reassuring, though caution is warranted in extrapolating these results to higher doses, longer durations, or populations with more severe iron deficiency. The inclusion of hepcidin, a key regulator of systemic iron homeostasis rarely assessed in EGCG trials, strengthens the safety assessment of this study.

Several methodological considerations merit discussion. First, blood samples were collected approximately 24 h after the last EGCG dose. Given EGCG's plasma half‐life of approximately 2–5 h, circulating EGCG concentrations at the time of sampling were likely negligible. Consequently, our biomarker measurements reflect cumulative, sustained effects of EGCG rather than acute peak‐concentration responses. Transient metabolic shifts in antioxidant capacity and acute catechin‐related metabolites, which may be most evident within 1–4 h of ingestion [52, 53, 54], would not have been captured by this sampling protocol, representing a potential underestimation of EGCG's short‐term bioactivity. Higher doses or longer durations may be required to elicit more pronounced effects, particularly in biomarkers with slower kinetics such as inflammatory cytokines and iron indices [18, 28, 36, 37, 38, 39]. Future studies should incorporate pharmacokinetically informed sampling time points to fully characterize acute and sustained metabolic effects.

### 4.1 Strengths and Limitations

This study had several important strengths. It is among the few clinical trials to simultaneously evaluate the effects of EGCG on lipid metabolism, inflammatory biomarkers, iron homeostasis, and untargeted plasma metabolomics within a single experimental design. The randomized, double‐blind, placebo‐controlled design with high adherence (> 90%) and confirmed blinding integrity enhanced internal validity. The inclusion of hepcidin provides a mechanistically relevant iron‐regulatory biomarker rarely assessed in EGCG trials. The absence of adverse events confirms the short‐term safety of EGCG supplementation at 400 mg/day in this population.

Limitations include the small sample size (n = 17), which precluded adequate statistical power and limited generalizability. The study enrolled exclusively White, non‐Hispanic women, limiting applicability to other demographic groups. The 8‐week intervention period may have been insufficient to detect changes in slower‐responding endpoints such as inflammatory cytokines. The untargeted metabolomic approach was exploratory and not validated by targeted assays. Blood sampling approximately 24 h post‐dose likely missed peak catechin concentrations and associated acute bioactivity. Compliance with caffeine and green tea abstinence relied on self‐reporting rather than objective biomarkers (e.g., urinary caffeine metabolites). Finally, dietary monitoring, while conducted via 3‐day food records, could not fully account for all sources of polyphenol intake or energy balance fluctuations.

## 5 Conclusion

This pilot RCT found no statistically significant effects of 8‐week EGCG supplementation (400 mg/day) on lipid profiles, inflammatory markers, or iron status in women with obesity; however, clinically directional trends in total and LDL cholesterol, ferritin, and hepcidin were noted. Untargeted metabolomics revealed exploratory pathway‐level shifts in lipid remodeling and β‐oxidation that may reflect systems‐level bioactivity of EGCG not captured by conventional endpoints. All metabolomic findings are hypothesis‐generating and require confirmation. Iron status was unaffected, supporting the short‐term safety of purified EGCG at this dose in this population. Future larger‐scale, longer‐duration RCTs incorporating validated targeted metabolomics, pharmacokinetically informed sampling, and objective dietary monitoring are needed to fully characterize the metabolic effects and dose–response relationships of EGCG supplementation in women with obesity.

List of metabolites with unadjusted p‐values < 0.05 from mixed‐effects models evaluating the interaction of treatment and time. These results did not retain significance after multiple testing corrections and were presented for an exploratory context.

## Author Contributions

All authors contributed to the critical review and final approval of the manuscript. **S.A**.: Assisted with study conception and design, participant recruitment, data collection, laboratory analysis, data analysis, interpretation, and manuscript drafting. **K.N.T**.: Metabolomics data processing, statistical analysis, and interpretation. **A.R.H**.: Study execution and laboratory support. **M.K**.: Study execution, laboratory analysis, and interpretation of findings. **M.R**.: Principal investigator, obtained funding, study conception, oversight of data collection, interpretation, and manuscript supervision.

## Funding

This research was supported internally by Doris A. Adams endowment chair funds from the Department of Food Science and Human Nutrition in the College of Human Sciences at Iowa State University. The funders played no role in the study design, data collection and analysis, decision to publish, or manuscript preparation.

## Ethics approval

The study protocol was approved by the Institutional Review Board of Iowa State University. Written informed consent was obtained from all participants prior to enrollment.

## Conflicts of Interest

The authors declare no conflicts of interest.

## Supporting information




**Supporting File**: mnfr70575‐sup‐0001‐TableS1.docx.

## Data Availability

The datasets generated and analyzed during the current study are available from the corresponding author upon reasonable request.
