# Supplementary material for: Effect of Epigallocatechin Gallate Supplementation on Inflammation, Iron Status, and Metabolomic Profiles in Women With Obesity: A Pilot Randomized Controlled Trial
Source: Mol Nutr Food Res. 2026 Aug 18;70(16):e70575. doi: 10.1002/mnfr.70575 (PMC13487353; doi:10.1002/mnfr.70575)
Supplement: Supplementary file 1 [file MNFR-70-e70575-s001.docx]

**Supplementary Table S1. Dietary intake of participants at baseline and after the 8-week intervention, by treatment group ^1^.**

| Dietary variable | Baseline | | | | Week 8 | | | |
| --- | --- | --- | --- | --- | --- | --- | --- | --- |
|  | **Total(n = 13)** | **Placebo(n = 8)** | **EGCG(n = 5)** | ***p*** | **Total(n = 14)** | **Placebo(n = 8)** | **EGCG(n = 6)** | ***p*** |
| Energy (kcal/d) | 2060.8 (795.1) | 2203.6 (709.9) | 1832.3 (953.1) | 0.44 | 1781.8 (427.9) | 1889.3 (339.7) | 1638.6 (521.0) | 0.30 |
| Protein (g/d) | 75.1 (29.5) | 81.2 (26.6) | 65.4 (34.3) | 0.37 | 61.9 (20.1) | 64.6 (23.9) | 58.3 (15.1) | 0.59 |
| Carbohydrate (g/d) | 256.9 (106.8) | 276.9 (101.0) | 224.8 (119.5) | 0.42 | 223.1 (70.5) | 227.8 (56.2) | 216.8 (91.7) | 0.78 |
| Fat (g/d) | 93.1 (35.9) | 103.6 (32.4) | 76.3 (38.3) | 0.20 | 69.9 (25.4) | 77.8 (29.8) | 59.5 (14.1) | 0.19 |
| Saturated fat (g/d) | 31.8 (11.4) | 34.3 (8.0) | 27.8 (15.7) | 0.34 | 26.0 (10.4) | 27.3 (12.0) | 24.2 (8.7) | 0.60 |
| PUFA (g/d) | 12.3 (8.1) | 14.5 (8.1) | 8.8 (7.5) | 0.22 | 8.0 (7.5) | 10.9 (8.6) | 4.2 (3.1) | 0.10 |
| Vitamin A (µg RAE/d) | 312.0 (220.5) | 391.7 (238.5) | 184.5 (114.9) | 0.10 | 492.5 (474.4) | 707.8 (520.1) | 205.5 (183.0) | 0.04 |
| Vitamin C (mg/d) | 36.5 (26.3) | 39.4 (20.5) | 31.8 (35.9) | 0.63 | 36.1 (43.0) | 54.8 (49.4) | 11.2 (8.7) | 0.06 |
| Vitamin D (µg/d) | 2.0 (1.9) | 2.8 (1.9) | 0.8 (1.2) | 0.06 | 2.9 (3.3) | 3.8 (4.0) | 1.6 (1.8) | 0.25 |
| Iron (mg/d) | 11.0 (5.1) | 12.3 (4.6) | 9.1 (5.8) | 0.29 | 11.1 (8.2) | 13.7 (10.0) | 7.8 (3.1) | 0.19 |

^1^ Values are mean (SD) of estimated daily intake derived from 3-day food records (two weekdays and one weekend day) at each timepoint; between-group comparisons (placebo vs. EGCG) used the independent *t* test. The number of participants (*n*) shown for each cell reflects only those who returned complete, analyzable 3-day records at that timepoint and therefore varies by group and timepoint (baseline: total 13 [placebo 8, EGCG 5]; week 8: total 14 [placebo 8, EGCG 6]). These counts are a subset of the fully randomized sample (placebo *n* = 9; EGCG *n* = 8). Dietary-record completion was independent of the clinical, biochemical, and metabolomic analyses, all of which retained the full randomized sample; no participant was excluded from the trial and the randomization pool is unchanged.

Comparisons were not adjusted for multiple testing. Intake was broadly comparable between groups; the nominally lower vitamin A intake in the EGCG group at week 8 (*p* = 0.04) should be interpreted cautiously given the small, unequal subsample sizes and the number of nutrients compared.

EGCG, epigallocatechin gallate; PUFA, polyunsaturated fatty acids; RAE, retinol activity equivalents.
